# Draft Genome Sequence of *Brevibacterium linens* AE038-8, an Extremely Arsenic-Resistant Bacterium

**DOI:** 10.1128/genomeA.00316-15

**Published:** 2015-04-16

**Authors:** Daniela Maizel, Sagar M. Utturkar, Steven D. Brown, Marcela A. Ferrero, Barry P. Rosen

**Affiliations:** a PROIMI-CONICET-Universidad Nacional de Tucumán, Tucumán, Argentina; b Graduate School of Genome Science and Technology, University of Tennessee, Knoxville, Tennessee, USA; c Biosciences Division, Oak Ridge National Laboratory, Oak Ridge, Tennessee, USA; d Department of Cellular Biology and Pharmacology, Herbert Wertheim College of Medicine, Florida International University, Miami, Florida, USA

## Abstract

To understand the arsenic biogeocycles in the groundwaters at Tucumán, Argentina, we isolated *Brevibacterium linens* sp. strain AE38-8, obtained from arsenic-contaminated well water. This strain is extremely resistant to arsenicals and has arsenic resistance (*ars*) genes in its genome. Here, we report the draft genome sequence of *B. linens* AE38-8.

## GENOME ANNOUNCEMENT

Arsenic is a toxic metalloid widely distributed in nature (1, 2) and ranks first on the U.S. government’s Priority List of Hazardous Substances because of its toxicity and prevalence in the environment (3). Arsenicals are introduced into the biosphere primarily from geologic formations, with some anthropogenic contributions as well (4, 5). Microorganisms have developed detoxification mechanisms that allow them to survive in high concentrations of arsenic (6). In Argentina, arsenic is a major public health concern, with millions of people exposed to arsenic in drinking water (7). The province of Tucumán is one of the most highly affected regions of the country, with high levels of arsenic in drinking water (8).

The goal of this study was to describe the arsenic biogeochemical cycle in the groundwater at Tucumán through an analysis of the genes and enzymes involved in arsenic metabolism. We sampled well water from Los Pereyra (N26°59′15.3″ W64°53′56.1″), an area known for its high content of arsenic in drinking water (up to 2 mg As/liter) (8). We enriched cultures of water samples in Luria Bertani (LB) medium amended with various concentrations of As(III) (arsenite) or As(V) (arsenate), resulting in the isolation of a strain of *Brevibacterium linens* that tolerates arsenicals at concentrations up to 1 M in 4-fold-diluted LB medium (data not shown); this is the first reported high-level-arsenic-resistant microbe found in Argentinian waters. Most previously described resistant microorganisms tolerate only much lower concentrations of As(III) or As(V) (9–12), while *B. linens* sp. strain AE038-8 is resistant to high concentrations of arsenicals and also biotransforms them, making this organism a candidate for future applications in arsenic bioremediation.

Genome sequencing was performed using an Illumina HiSeq platform, with quality-based trimming, as described previously (13). After trimming, 6,211,868 paired-end reads remained, with an average length of 90 bp, comprising a genome coverage of 146×. After evaluating several approaches (14), the optimal assembly was obtained using the SPAdes software (version 3.1.1), which consisted of 29 large (≥500 bp) contigs, with a total genome size of 3.8 Mb. Gene prediction and annotation were performed at the Oak Ridge National Laboratory, as described previously (15). The draft genome sequence has an overall G+C content of 64.2%, *N*_50_ contig of 327 kb, a largest contig of 831 kb, and contains 3,434 candidate protein-coding genes. The genes for putative arsenic resistances identified in this genome include an *ars* operon containing *ACR*3 [encoding for a membrane efflux pump responsible for the extrusion of As(III) outside the cell] (16), *ACR*2 (encoding a cytosolic arsenate reductase) (17), and *ars*R [encoding an As(III)-responsive repressor protein] (18), as well as a putative *arsM* gene [encoding for an *S*-adenosyl-methionine (SAM)-dependent methyltransferase that might methylate As(III)] (6). In summary, this extremely arsenic-resistant strain may have future usefulness in arsenic bioremediation.

### Nucleotide sequence accession number. 

The draft genome sequence of strain AE038-8 has been deposited at DDBJ/EMBL/GenBank under the accession no. JTJZ00000000. The version described in this paper is the first version.
